# Insights into the skin of caecilian amphibians from gene expression profiles

**DOI:** 10.1186/s12864-020-06881-8

**Published:** 2020-07-27

**Authors:** María Torres-Sánchez, Mark Wilkinson, David J. Gower, Christopher J. Creevey, Diego San Mauro

**Affiliations:** 1 Department of Biodiversity, Ecology and Evolution, Complutense University of Madrid, 28040 Madrid, Spain; 2 Present address: Department of Biology, University of Florida, Gainesville, Florida, 32611 USA; 3 Department of Life Sciences, The Natural History Museum, London, SW7 5BD UK; 4 Institute for Global Food Security, Queen’s University Belfast, University Road, Belfast, Northern Ireland BT7 1NN UK

**Keywords:** Bioactive peptides, Claudins, Cornified proteins, Gymnophiona, Keratins, Transcriptomics

## Abstract

**Background:**

Gene expression profiles can provide insights into the molecular machinery behind tissue functions and, in turn, can further our understanding of environmental responses, and developmental and evolutionary processes. During vertebrate evolution, the skin has played a crucial role, displaying a wide diversity of essential functions. To unravel the molecular basis of skin specialisations and adaptations, we compared gene expression in the skin with eight other tissues in a phylogenetically and ecologically diverse species sample of one of the most neglected vertebrate groups, the caecilian amphibians (order Gymnophiona).

**Results:**

The skin of the five studied caecilian species showed a distinct gene expression profile reflecting its developmental origin and showing similarities to other epithelial tissues. We identified 59 sequences with conserved enhanced expression in the skin that might be associated with caecilian dermal specialisations. Some of the up-regulated genes shared expression patterns with human skin and potentially are involved in skin functions across vertebrates. Variation trends in gene expression were detected between mid and posterior body skin suggesting different functions between body regions. Several candidate biologically active peptides were also annotated.

**Conclusions:**

Our study provides the first atlas of differentially expressed sequences in caecilian tissues and a baseline to explore the molecular basis of the skin functions in caecilian amphibians, and more broadly in vertebrates.

## Background

Most nucleated cells of a multicellular organism contain the same genetic information, with the functional diversity of different organs and tissues mainly caused by differences in gene expression and regulation. Gene expression profiles of a tissue of an organism may vary with ontogeny and in response to environmental conditions, with differences between tissues reflecting their specialised functions [1, 2]. Tissue-specific gene expression profiles are also expected to differ among species, underpinning interspecific phenotypic variation. Comparisons of these profiles should provide insight into the evolutionary history of diversification and adaptation [3, 4]. Data on tissue-specific gene expression have been massively boosted by the application of high throughput sequencing (HTS) technologies in, mainly, epigenomics, transcriptomics, and proteomics.

The outer tissue of vertebrate animals, the skin, is formed by multiple cell layers that are in the frontline of direct interactions with both abiotic and biotic components of the environment. The skin is one of the largest organs in vertebrates, and is involved in multiple vital functions including protection, defence, communication, and reproduction. This functional diversity is coupled with various specialised structures in different taxa, including glands, pigment cells, scales, claws, horns, feathers and fur [5, 6]. In amphibians, the skin is often a moist, relatively thin, permeable tissue with keratinisation mainly in the outermost cell layer (stratum corneum) of the epidermis and with diverse exocrine glands distributed in the dermis [7, 8]. Mucous glands play an important role in transitions between aquatic and terrestrial environments by helping to prevent dehydration [9]. Mucus also facilitates cutaneous respiration [10], locomotion and escape from predators [7, 11]. Granular glands can produce cocktails of diverse biologically active compounds, some of which are essential for defence and communication [12, 13], such as antimicrobial peptides (AMPs) and pheromones.

Caecilians (order Gymnophiona) are relatively poorly known, snake-like, mainly tropical amphibians. Adults of most caecilian species live in soils and features associated with their fossorial lifestyle include elongate limbless bodies, reduced eyes and a pair of sensory tentacles on the snout [14, 15]. Caecilian skin is annulated, with dermal folds that, in many species, hide dermal scales, a unique specialisation among extant tetrapods the function of which is not well-understood [16–18]. In some caecilian species, the maternal skin periodically stores lipid reserves to provide nutrition to dermatophagous hatchlings [19–21].

Molecular characterisation of caecilian skin features and functions is scarce. Preliminary studies of multi-tissue transcriptomes from five species of caecilians identified candidate novel gene families with skin-specific expression [22], and a skin collagen gene (*col17a1*) that has been under positive selection in the branch subtending the studied species [23]. Given the distinctiveness of caecilian skin, a thorough characterisation of tissue-specific gene expression profiles could reveal the molecular machinery behind skin functions that potentially played a crucial role in the adaptation of the group. To identify genes with conserved expression patterns in the skin across the five studied caecilian species (*Rhinatrema bivittatum* [Guérrin-Méneville, 1838], *Caecilia tentaculata* Linnaeus, 1758, *Typhlonectes compressicauda* [Duméril & Bibron, 1841], *Microcaecilia unicolor* [Duméril, 1861] and *Microcaecilia dermatophaga* Wilkinson, Sherratt, Starace & Gower, 2013), we conducted a differential expression analysis comparing caecilian skin samples to other tissue samples, and annotated skin bioactive peptides using the same multi-tissue caecilian transcriptomic data.

## Results

**Fig. 1:**
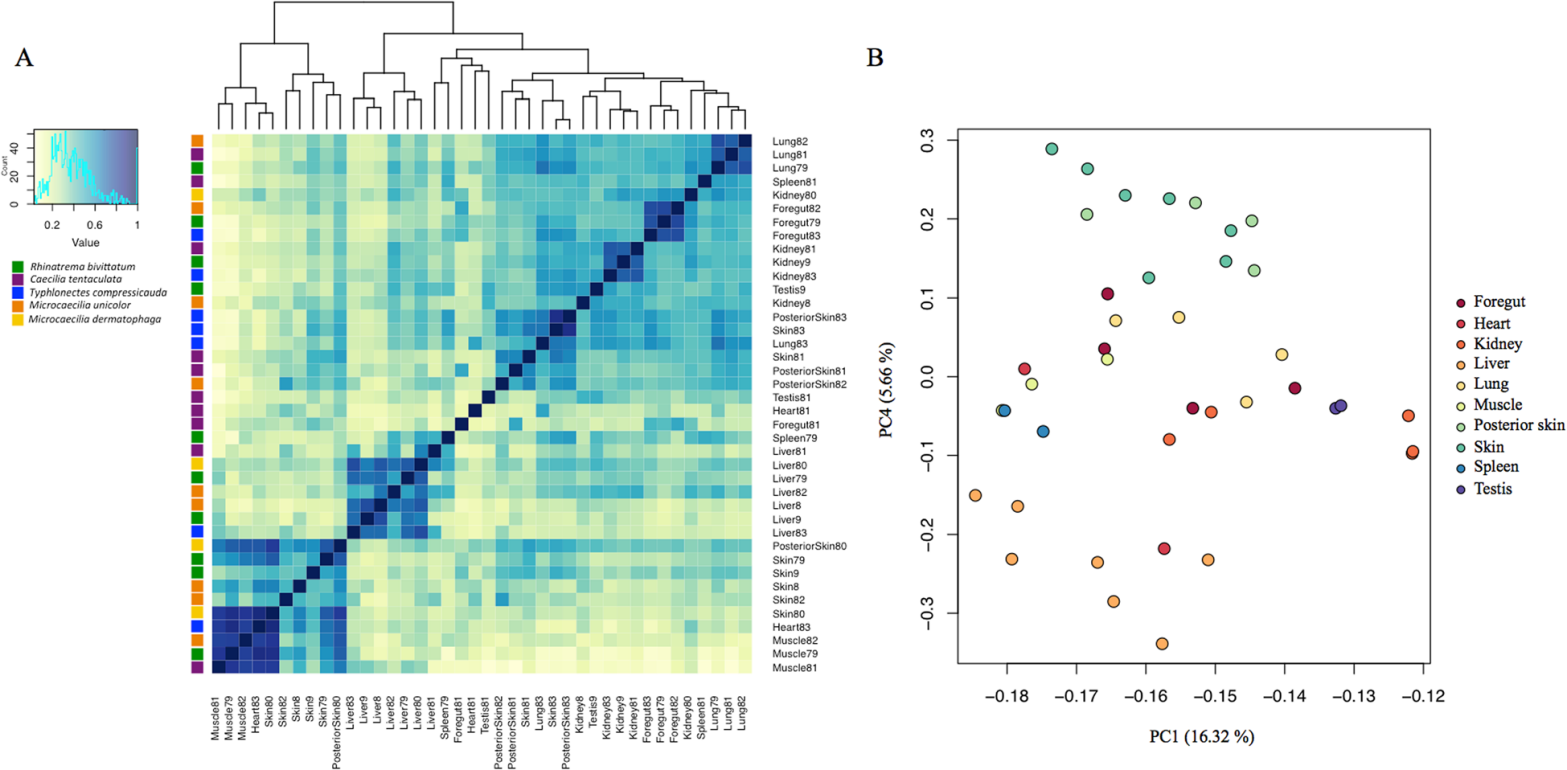
Gene expression variation across samples of nine tissue types in seven individuals of five caecilian species. **a** Heatmap showing correlation between variance-mean expression levels for protein-coding genes in different tissue samples (see Table S2 for sample details). **b** PCA plot of PC1 versus PC4 showing variance among gene expression levels in various tissue types across the five sampled caecilian species

A total of 2624 protein-coding sequences shared identical UniProt best-hit annotations across the five caecilian transcriptomes. Variance-means correlations and hierarchical clusters among tissue samples from the gene expression levels of these 2624 sequences are shown in Fig. 1a. Samples partially clustered by tissue type, indicating gene expression correlation across the five species. Two groups of samples with closely correlated gene expression levels were identified: (i) *R. bivittatum* (midbody Skin9 and midbody Skin79), *M. unicolor* (midbody Skin8 and midbody Skin82), and *M. dermatophaga* (midbody Skin80 and posterior region PosteriorSkin80) samples; and (ii) *M. unicolor* (posterior region PosteriorSkin82), *C. tentaculata* (midbody Skin81 and posterior region PosteriorSkin81)*,* and *T. compressicauda* (midbody Skin83 and posterior region PosteriorSkin83) samples. Both of these skin groups were however clustered with samples from other tissue types. The first skin group was found in two related clusters, in which the *M. dermatophaga* midbody sample (Skin80) was more similar to muscle tissues (cardiac: Heart83, and skeletal: Muscle81, Muscle79 and Muscle82). The second skin group was clustered with the lung sample from *T. compressicauda* (Lung83) that was highly correlated with both of the skin samples of the same species (Skin83 and PosteriorSkin83). Samples were classified also by the decomposition of their variance with the first six principal components (PCs) of the PCA together explained 45.15% (= 16.32 + 6.67 + 6.33 + 5.66 + 5.16 + 5.01) of the total variance of the gene expression, and with each subsequent component explaining less than 5% of the expression variance. The first three components grouped the samples per species (Fig. S1). Skin samples were clustered toward positive values of PC4 (Fig. 1b). Lung and foregut samples were the most similar tissue samples to skin along PC4, and liver samples the most dissimilar, distributed toward high negative values along PC4.

**Fig. 2:**
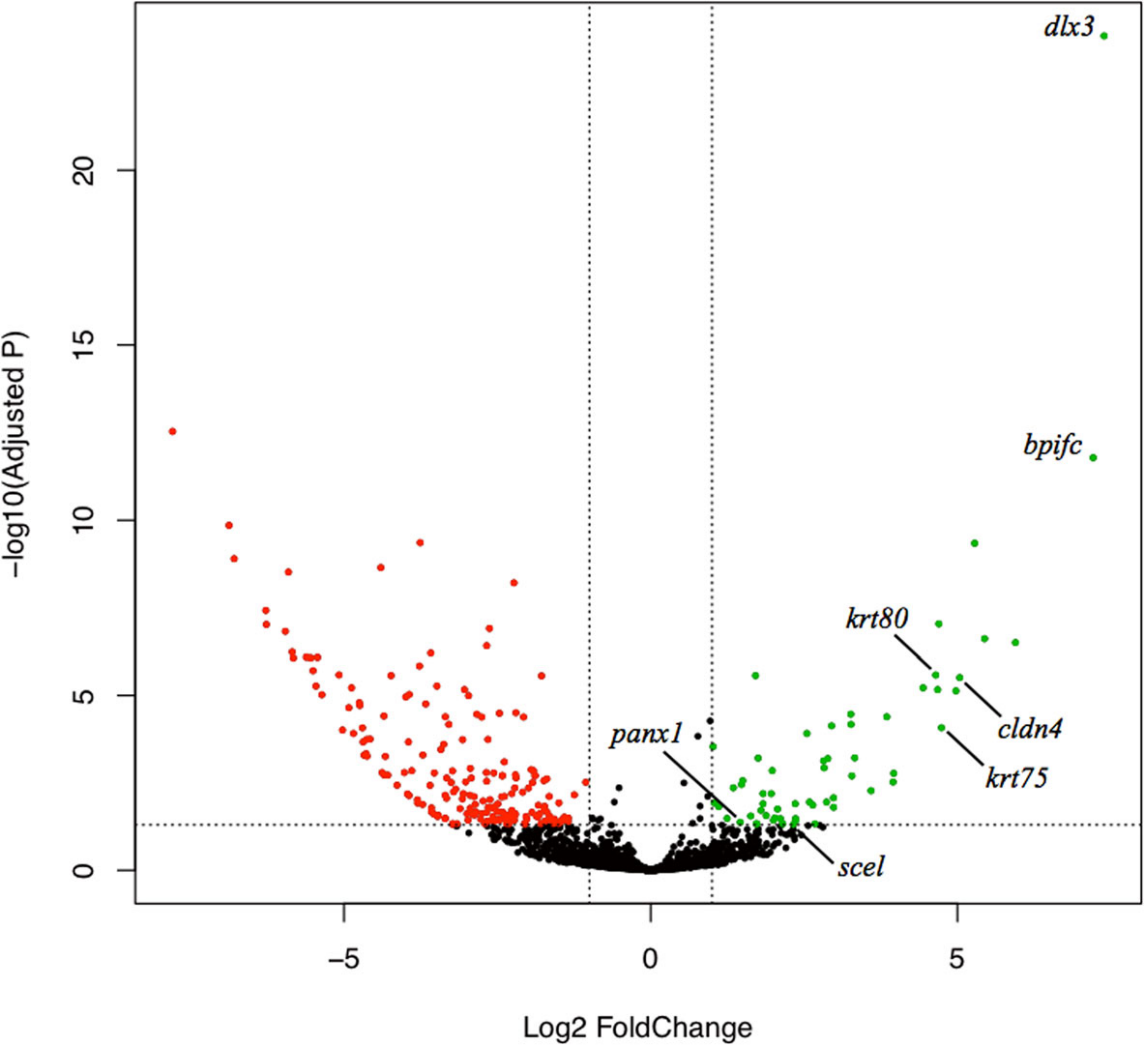
Protein-coding genes differentially expressed in caecilian skin. The plot shows the magnitude of difference in expression levels between skin and non-skin tissues, with red dots indicating significantly down- and green dots significantly up-regulated genes. Sequences identified as differentially expressed in the skin were those with one logarithmic unit of fold change difference in variance-mean between skin (11 replicates: midbody + posterior skin samples from the different caecilian species) and non-skin tissues (29 samples) with adjusted *p*-values < 0.05

We identified 246 sequences with differential expression in caecilian skin in the analysis comprising the 40 tissues (see Fig. 2 and Additional file 2). Among these sequences, 59 were identified as up-regulated or skin enriched sequences (see Fig. 2 and Table S3) with 12 of these having a positive fold change greater than four units (*atp13a4, bpifc, cldn4, dlx3, fat2, krt75, krt80, pou3f1, plca, tfap2c, tfap2e,* and *znf750*). These sequences were found also up-regulated with high fold change in the analysis using only tissues from the same sequencing company, while down-regulated sequences varied depending on the tissues included in the differential expression analysis (see Additional file 3). Because down-regulated sequences in the skin depended on the tissues included in the contrast, we focus on the more consistent results of the up-regulated sequences. Seventeen of the identified up-regulated caecilian skin genes (*abca12, ankk1, bpifc, dlx3, dsc1, esyt3, fam212a, fat2, gjb5, krt80, pou3f1, scel, tfap2c, tfap2e, tgm5 wnt7b,* and *znf750*) have enhanced expression in human skin. Three up-regulated caecilian skin genes (*adgrg6, dlx3, fam26d*) have enriched expression in human placenta. A total of 11 up-regulated caecilian skin sequences had a higher mean expression in posterior than midbody skin samples (*clnd4:* posterior skin mean expression = 2186.75 and midbody skin mean expression = 1852.9, *plca, fat2, abca12, tmprss4, mgat5b, tnfrsf16, qnr-71, hlf, wdr47,* and *ahnak*). The GO terms for the skin up-regulated genes (Table S3) were summarized and visualized in network graphs (Fig. S2). In addition to constitutive cellular processes, the skin up-regulated sequences were annotated with biological process terms related to epidermis development (GO:0008544, GO:0031424), epithelial cell migration (GO:0010631), cornified envelops (GO:0001533), circadian rhythm (GO:0007623), and pathogenesis and secretion (GO:0005576), among others. Binding functions were the predominant molecular functions annotated for the skin up-regulated genes that were operating in different cell compartments according to GO term annotations. The enrichment analysis found no evidence of protein-protein interactions (*p*-value = 0.0947) for the skin up-regulated genes.

A total of 134 protein-coding sequences expressed in the skin of at least one of the five caecilian species were annotated as chemical peptides, being either AMPs or peptide pheromones (91 candidate AMPs and 43 candidate peptide pheromones: 11 in *R. bivittatum*, 11 in *C. tentaculata*, 10 in *T. compressicauda*, 12 in *M. unicolor* and 12 in *M. dermatophaga*; for further detail see Tables S4–6). The vast majority of the candidate peptide pheromones were annotated as sodefrin precursor-like factors (SPF) except one protein-coding gene expressed in the skin of *C. tentaculata* that was annotated as aphrodisin (Table S5). Approximately 30% of the candidate protein-coding sequences encoding AMPs were expressed in the skin of all five sampled caecilian species (28 candidate AMPs, see Fig. S3 and Table S4). The remaining 70% candidate AMPs were either species specific or shared by subsets of the five study species. In contrast, none of the candidate protein-coding sequences encoding peptide pheromones was shared among the five sampled species, with more than 80% of the candidate peptide pheromones being species specific (35 candidate peptide pheromones, see Fig. S3 and Table S5). AMP gene expression was significantly higher in posterior than midbody skin for the four species for which this comparison was possible (Table S6). Peptide pheromone gene expression showed no significant differences between midbody and posterior skin expression values.

## Discussion

### General expression patterns in caecilian skin

Gene expression profiles are complex phenotypic traits that can reflect many biological phenomena including responses to the environment, development and differentiation processes of tissues and organs, and selective pressures and evolutionary histories of species [1–4]. In this study, we analysed a subset of the gene expression profiles of several tissues from five phylogenetically and ecologically diverse caecilian species. That less than half of the variance of gene expression in the caecilian tissue samples was captured by the first six principal components of our analysis, with each PC explaining a small percentage of the total variance, might be partly explained by the inclusion of 40 transcriptomic samples comprising nine different tissue types from seven individuals of the five studied caecilian species. Despite this heterogeneity, general tissue variation patterns were obtained through the decomposition of the total variance. While the first components grouped the samples by species as a result of the variance assessment across the dataset as a whole [24], gene expression variance along PC4 separated samples by tissues and could be accounted for by differences in embryonic germ layers and subsequent differentiation [25]. Skin samples that have epithelial and mesenchymal components and that are representative of ectoderm and mesoderm derived tissues were clearly distinct in gene expression from liver samples that have an endodermal origin. The most similar mesodermal and endodermal samples to the skin, in terms of gene expression variance along PC4, were lung and foregut samples, both of which resemble skin in having an epithelium that interacts directly with the environment. Although technical variation between RNA-seq samples is usually smaller than biological variation [24], the tissue distinction described here could have been magnified by technical differences between the sequencing companies.

Caecilian skin also showed a different expression pattern than other tissue types based on the total sample variance. Skin samples were grouped in two clusters with some degree of potential phylogenetic signal: samples from the sister families Caeciliidae and Typhlonectidae (*C. tentaculata* and *T. compressicauda,* respectively) were correlated. Beyond possible phylogenetic signal, skin samples also clustered by body region, as exemplified by the midbody and posterior region skin samples of *M. unicolor*. Gene expression in posterior skin of *M. unicolor* was more closely correlated with posterior skin samples of other taxa (*C. tentaculata* and *T. compressidauca* samples) than with midbody skin of the same specimen. These expression data are consistent with histological studies documenting regional differences in skin morphology and the relative abundances of mucous and granular glands in caecilians [26], supporting the hypothesis of regional differential function as well as form.

Some gene expression correlations with other tissue types were found for the identified skin clusters. Clustering of skin of *M. dermatophaga* with muscle samples might be explained by contamination of skin samples with stray muscle fibres due to the small size of this species and the difficulty of separating skin from the muscle during rapid dissection. Clustering of *T. compressicauda* lung (Lung83) with skin samples from the same species was different to the pattern for lung and skin of other species. In this case, contamination is less likely for these non-adjacent tissue types, and this result might instead be explained by some difference in skin and/or lung function in this species. It might be noted that *T. compressicauda* is the only sampled species that is fully aquatic as adults. Although *T. compressicauda* has the largest known lungs of any caecilian species [27], its skin might also perform a respiratory function and could have special gene expression patterns to perform gas exchange in aquatic environments [28]; possibly analogous to the skin expression pattern of plethodontid salamanders where a surfactant protein, expressed normally in the lungs, has been identified [29].

### Skin up-regulated genes

That some sequences were up-regulated in both caecilians and humans is perhaps indicative of highly conserved gene expression patterns and skin function across tetrapod vertebrates. Among the 12 caecilian skin up-regulated sequences with the highest differential expression values, eight genes have enhanced expression also in human skin: *dlx3* (distal-less homeobox 3), *bpifc* (bactericidal/permeability-increasing protein), *znf750* (zinc finger protein 750), *fat2* (protocadherin Fat2), *pou3f1* (POU class 3 homebox 1)*, tfap2c* (transcription factor AP-2 gamma)*, tfap2e* (transcription factor AP-2-epsilon)*,* and *krt80* (keratin 80). In mammals, *dlx3* has a crucial role, among others, in the differentiation of hair follicles [30]. Amphibian skin is extremely rich in diverse glands, and we hypothesize that *dlx3* in caecilians might be involved in the differentiation of cutaneous glands and dermis development. Similarly, *znf750* and *pou3f1* have been reported as compelling transcriptional regulators of epidermal cell differentiation in humans [31, 32] and both could play a similar role in caecilians. The other two transcription factors with enhanced expression in both caecilian and human skin (*tfap2c* and *tfap2e*) could also contribute to this process. Epidermal differentiation culminates in the emergence of specialised cells such as keratinocytes that are the most abundant skin cell type in many vertebrates, especially in the outermost layer of the epidermis where these cells form the first physical barrier of the skin [33]. Many of the identified up-regulated genes in caecilian skin were related to keratinocyte biology, from cell formation to migration process. In keratinocytes, diverse keratins and also cadherins are produced [34], and their encoding genes display up-regulation, which is in line with the high skin expression levels for *krt80, fat2* and *krt75* identified in our study. Keratins are a family of fibrous proteins involved in cornification, the main function of which is epithelial protection from external damage and stress [35]. The skin’s barrier function is also highlighted in humans by up-regulation of *bpifc* [36], which is perhaps also part of the general antibacterial defence mechanism of caecilian skin. Other up-regulated sequences common to humans and caecilians include *scel* (sciellin), a precursor of cornified structures that is expressed also, for example, in the shell of soft-shelled turtles [37]. This cornification protein perhaps helps effect the barrier function of caecilian skin, likely important during locomotion within soil.

In contrast to the genes with enhanced expression in both caecilian and human skin, we found caecilian skin sequences that are generally expressed in all human tissues or with enhanced expression in non-skin tissues, such as placenta. Two such sequences among the highest differentially expressed genes in caecilian skin are *cldn4* (claudin 4) and *atp13a4* (probable cation-transporting ATPase 13A4). Claudins are transmembrane proteins, crucial components of cellular tight junctions that constitute a second barrier beneath the outer keratinised epidermis preventing, for example, uncontrolled loss of water through the skin [38]. In our analysis, this up-regulated gene showed greater expression in posterior skin, where mucous glands tend to be less and granular glands more abundant [26]. Lower mucous secretion in the posterior skin of caecilians might result in hydric stress. Electrolyte homeostasis and fluid balance in caecilian skin could also be affected by the transmembrane ATPase encoded by *atp13a4*. These two genes might, potentially, have been involved in the adaptation to terrestrial environments in caecilians, as well as other amphibians.

Three up-regulated caecilian skin sequences, *dlx3* (also up-regulated in human skin)*, fam26d* (family with sequence similarity 26 member D), and *adgrg6* (adhesion G-protein coupled receptor G6) are also up-regulated in the human placenta, the organ that provide nutrients to eutherian foetuses [39–41]. During skin feeding in caecilians, maternal epidermis is nutritionally enriched (hypertrophied and lipidified) and the stratum corneum is episodically removed by hatchlings with the help of a specialized vernal dentition, requiring repeated rematuration of the outer layers of the epidermis [19, 20]. In caecilian skin, *dlx3*, *fam26d*, and *adgrg6* might be potentially associated with this epidermal nourishment that many species, including some of those studied here, provide to their offspring. Nevertheless, these three genes were expressed in all the studied species without a clear trend of major enhanced expression in the skin feeders, which also were not in the epidermal nourishment life stage when skin sampling for RNA extraction occurred [22]. Other caecilian skin up-regulated sequences with no enhanced expression in human tissues include *panx1* (pannexin 1), which is involved in skin regeneration [42], and which might have the same role in caecilians, helping with the reconstitution of the skin after injury, skin-feeding events, and/or normal shedding cycles.

### Chemical defence and communication underground?

Biologically active peptides are essential for many taxa, including amphibians, which are well known for their ability to synthesise diverse AMPs and peptide pheromones [7]. Although many bioactive peptides have been characterised in frogs and salamanders, knowledge about this type of peptide in caecilians is scarce. We annotated several candidate sequences expressed in the caecilian skin that could encode AMPs and SPF peptide pheromones. SPF proteins identified here in caecilians belong to the same gene family of sodefrin that is a courtship pheromone produced in the gonads of male salamanders [43, 44]. Our results indicate the potential production of a multiple pheromone cocktail with high species-specificity in both male and female caecilians. Male and female pheromone production is perhaps an adaptation to finding mates in animals that have reduced visual systems.

Unlike peptide pheromones, many candidate AMPs were annotated across the sampled caecilian species denoting, plausibly, a general common chemical defense system across caecilians. Several of the AMP annotations were previously known only from other animal groups. Magainins and andersonins have been reported in various frog lineages [45, 46] and cecropins are found exclusively in insects [47]. The presence of these candidate AMP genes in caecilian amphibians potentially suggests convergent evolution, but it must be stressed that some candidate AMP sequences might have resulted from misidentifications given that our annotations are reliant only on similarity searches, for which error rates are higher when annotating small molecules such as AMPs. A greater prevalence of candidate AMP expression in posterior skin samples is consistent with the higher abundance of toxin secreting granular glands in the posterior body region of caecilians [26].

## Conclusions

We identify general patterns of gene expression that highlight genes that are potentially involved in skin functions across vertebrates as well as those likely related to special features of caecilian skin. Further studies are required to provide finer resolution, better gene annotation and accurate function, and to more thoroughly explore gene expression related to the diverse morphology, ecology, and evolutionary history of caecilians. This study provides baseline information about the molecular biology of caecilian skin, and will hopefully promote further studies into skin function and adaptation during vertebrate evolution.

## Methods

The source data for this study were the protein-coding sequences from five reference species-specific caecilian transcriptomes, and the raw sequence reads of 40 tissue samples (data are available to download from the NCBI through BioProject PRJNA387587, SRA database accession numbers for each tissue sample are also provided in Table S2), that were paired-end sequenced on Illumina HiSeq2000 by two different companies after poly-A enrichment and TruSeq library preparation [22]. Tissues were collected from freshly sacrificed specimens, which were captive maintained (but wild-caught in French Guiana) under controlled conditions. After anaesthetising the animals with tricaine methanesulphonate (MS222), tissue samples were mechanically separated and immediately soaked in RNAlater stabilization solution [22]. In the case of the skin samples, after initial removal the internal surface of the skin was examined for any adhering muscle fibres or connective tissue which were mechanically removed with forceps or scrapped off with a scalpel under a dissection microscope. For each species (*R. bivittatum*, *C. tentaculata*, *T. compressicauda*, *M. unicolor* and *M. dermatophaga*), the transcriptome was built by pooling the separate sequenced RNA samples from multiple tissues: skin (midbody skin tissue samples for all five species and posterior skin samples for four species), liver, lung, kidney, foregut, testis, heart, spleen, and muscle (see Table S2 for sample details and experimental design; for more details about the transcriptome reconstructions see [22]). To characterise caecilian skin expression profiles, we conducted differential gene expression analysis, and annotated the transcriptomic sequences to identify sequences that putatively encode candidate AMPs and peptide pheromones. Features of the ecology and skin of the five studied species are summarised in Table S1.

Protein-coding sequences of the five species-specific caecilian transcriptomes were annotated against the reviewed protein database of UniProt, Swiss-Prot [48] by using the blastp tool of BLAST 2.2.28 [49] and retrieving output records with an e-value threshold of 1e-20. Only annotated sequences with identical best hits across all the species-specific transcriptomes were used in further analyses. Gene expression levels of each gene in each sample were estimated using HTSeq 0.6.1 [50] after mapping the reads to their assembly with Bowtie 2.0.2 [51]. Raw expression counts per sample were multiplied by the mean count across their species and divided by the mean count of all 40 samples, in order to scale expression values per each species.

Variance-mean estimates from scaled expression counts were calculated for each sample after normalisation based on a negative binomial distribution using the Bioconductor package DESeq2 [52]. To explore the general gene expression patterns from all tissue samples, these data were subjected to principal components analysis (PCA) and hierarchical cluster analysis using the function princomp of R 3.3.0 [53]. Conserved skin gene expression patterns across caecilian species were identified by a differential expression analysis between skin and non-skin samples (as a baseline of expression) using also DESeq2 [52]. Sequences identified as differentially expressed in the skin were those with one logarithmic unit of fold change difference in variance-mean between skin (11 replicates: midbody + posterior skin samples) and non-skin tissues (29 samples) with adjusted *p*-values < 0.05.

In order to assess whether technical differences between the sequencing companies (BGI and Macrogen; [22]) could be biasing the results, differential expression analyses including only samples from the same company (skin tissues vs non-skin tissues: foregut, kidney, and lung) were also performed, retrieving the same results for the majority of the up-regulated sequences. We obtained gene ontologies (GOs) for the identified sequences that were expressed differentially in the skin using the UniProt Retrieve/ID mapping tool [48]. GO terms and adjusted *p*-values of the differential expression analysis were summarized and visualized using REVIGO [54] with 0.4 of allowed semantic similarity (threshold that reduced at maximum the list of terms; more conservative thresholds were explored to ensure that not redundant terms were removed) and the entire UniProt database defining the GO term size (number of UniProt genes annotated for each term). Protein-protein interactions (PPis) and functional enrichments for differentially expressed sequences with a positive fold change in the skin (up-regulated sequences) were sought using STRING [55] with default parameters. Skin up-regulated sequences were also queried against the Human Protein Atlas dataset [56] to contrast expression levels and tissue specificity in humans. The scaled expression values of the identified differentially expressed sequences were further explored by classifying the 11 skin samples by skin region: posterior (4 samples) versus midbody (7 samples) and comparing their mean expression values, in order to seek expression differences between these body regions.

AMP annotation of sequences expressed in skin used similarity searches against three datasets: ADP3 database [57], DADP database [58], and a self-built database containing andersonin, cathelicidin, cecropin, and magainin sequences from the UniProt database [48], using the blastp tool of BLAST 2.2.28 [49] and retrieving output hits with an e-value threshold of 1e-5. Pheromone annotation for sequences expressed in skin was performed also by the same similarity search strategy against another self-built database containing sodefrin, splendipherin, and aphrodisin sequences from the UniProt database [48]. We tested the null hypothesis of no difference in gene expression levels of the candidate AMPs and peptide pheromones between midbody and posterior skin samples using transcripts per million (TPM) expression values calculated with RSEM [59], and by applying Wilcoxon signed-rank tests with R 3.3.0 [53]. We evaluated the commonality of the specific candidate AMP and peptide pheromones by identifying the same annotated hit among the sampled caecilian species.

## Supplementary information

**Additional file 1: Table S1.** Summary of variation in ecology and skin morphology for the five sampled caecilian species. Dermal fold (annulation) system categories as follows: primary = borders between folds aligned with those between underlying somites and vertebrae; secondary = folds midway between borders underlying somites and vertebrae; “tertiary” = many folds, not aligned with underlying somites and vertebrae (see Gower and Wilkinson [2007], and Wilkinson and Nussbaum [17]. Degree of fossoriality (see Bardua et al. [2019]) and habitat is based on observation in the field (DJG, DSM, MW pers. obs.). See San Mauro et al. [21], Taylor [14], and Wilkinson, Sherratt, Starace, and Gower (2013) for more information on morphology and maternal dermatophagy. **Table S2**. Tissue sample information and experimental design for the differential expression analysis. **Table S3**. Characterisation of significantly up-regulated sequences in the caecilian skin transcriptomes. **Table S4.** Antimicrobial peptide annotation (APD31, DADP2, Uniprot terms: Andersonin3, Cathelicidin4, Cecropin5 and Magainin6) and occurrence (indicated by an X) in skin transcriptomes of the five sampled caecilian species. **Table S5.** Peptide pheromones annotation and occurrence (indicated by an X) in skin transcriptomes of the five sampled caecilian species. **Table S6**. Annotated AMPs and *p*-values for Wilcoxon signed-rank test of differences between AMP expression levels in midbody and posterior skin samples. * indicates selfbuilt databases for subset of entries for these UniProt terms (see Materials and Methods). For *R. bivittatum,* data were available only for midbody skin. **Figure S1**. PCA plots of PC1 versus PC2 (first column), PC2 versus PC3 (second column), and PC1 versus PC4 (third column) showing variance among gene expression levels in various tissue types across the five sampled caecilian species. Samples were color coded by tissue type (first row), species (second row), and sequencing company (third row). **Figure S2**. Network graphs for GO domains (A: biological process, B: molecular function and C: cellular component) of skin up-regulated genes. Greater colour intensity indicates higher fold change in expression in caecilian skin, and circle size is positively correlated with number of genes with the same GO in the UniProt database. **Figure S3**. Expressed sequences annotated as encoding peptides and their presence in the skin of the five sampled caecilian species.

**Additional file 2 **Table with the differential expressed sequences with adjusted *p*-values < 0.05 and one logarithmic unit of fold change difference in variance-mean between skin and non-skin samples.

**Additional file 3.** Table with the results of the differential expression analysis including only samples from the same company.

## Data Availability

Tissue-specific RNA-seq reads and species-specific de novo transcriptome assemblies are available from NCBI through BioProject ID number PRJNA387587 (SRA database accession numbers for each tissue sample are also provided in Table S2).

## Abbreviations

AMPs: Antimicrobial peptides
GO: Gene ontology
HTS: High throughput sequencing
PCA: Principal components analysis
PCs: Principal components
PPis: Protein-protein interactions
SPF: Sodefrin precursor-like factor
TPM: Transcripts per million
