# Supplementary material for: Insights into the skin of caecilian amphibians from gene expression profiles
Source: BMC Genomics. 2020 Jul 27;21:515. doi: 10.1186/s12864-020-06881-8 (PMC7385959; doi:10.1186/s12864-020-06881-8)
Supplement: Supplementary file 1 — Additional file 1: Table S1. Summary of variation in ecology and skin morphology for the five sampled caecilian species. Dermal fold (annulation) system categories as follows: primary = borders between folds aligned with those between underlying somites and vertebrae; secondary = folds midway between borders underlying somites and vertebrae; “tertiary” = many folds, not aligned with underlying somites and vertebrae (see Gower and Wilkinson [2007], and Wilkinson and Nussbaum [17]. Degree of fossoriality (see Bardua et al. [2019]) and habitat is based on observation in the field (DJG, DSM, MW pers. obs.). See San Mauro et al. [21], Taylor [14], and Wilkinson, Sherratt, Starace, and Gower (2013) for more information on morphology and maternal dermatophagy. Table S2. Tissue sample information and experimental design for the differential expression analysis. Table S3. Characterisation of significantly up-regulated sequences in the caecilian skin transcriptomes. Table S4. Antimicrobial peptide annotation (APD31, DADP2, Uniprot terms: Andersonin3, Cathelicidin4, Cecropin5 and Magainin6) and occurrence (indicated by an X) in skin transcriptomes of the five sampled caecilian species. Table S5. Peptide pheromones annotation and occurrence (indicated by an X) in skin transcriptomes of the five sampled caecilian species. Table S6. Annotated AMPs and p-values for Wilcoxon signed-rank test of differences between AMP expression levels in midbody and posterior skin samples. * indicates selfbuilt databases for subset of entries for these UniProt terms (see Materials and Methods). For R. bivittatum, data were available only for midbody skin. Figure S1. PCA plots of PC1 versus PC2 (first column), PC2 versus PC3 (second column), and PC1 versus PC4 (third column) showing variance among gene expression levels in various tissue types across the five sampled caecilian species. Samples were color coded by tissue type (first row), species (second row), and sequencing company (third row). Fig [file 12864_2020_6881_MOESM1_ESM.pdf]

SUPPLEMENTARY MATERIAL

Insights into the skin of caecilian amphibians from gene expression profiles

María Torres-Sánchez[1,2,*], Mark Wilkinson[3], David J. Gower[3], Christopher J. Creevey[4], Diego San Mauro[1]

[1]Department of Biodiversity, Ecology and Evolution, Complutense University of Madrid, 28040 Madrid, Spain

[2]Present address: Department of Neuroscience, Spinal Cord and Brain Injury Research Center & Ambystoma Genetic Stock Center, University of Kentucky, Lexington, KY, 40536, United States

[3]Department of Life Sciences, The Natural History Museum, London SW7 5BD, UK

[4]Institute for Global Food Security, Queen's University Belfast, University Road, Belfast, BT7 1NN, Northern Ireland, UK.

***Author for Correspondence**: torressanchez.maria@gmail.com

This file includes:

Tables S1–S6

Figure S1

Figure S2

Figure S3

Table S1. Summary of variation in ecology and skin morphology for the five sampled caecilian species. Dermal fold (annulation) system categories as follows: primary = borders between folds aligned with those between underlying somites and vertebrae; secondary = folds midway between borders underlying somites and vertebrae; “tertiary” = many folds, not aligned with underlying somites and vertebrae (see Gower and Wilkinson [2007], and Wilkinson and Nussbaum [2006]. Degree of fossoriality (see Bardua et al. [2019]) and habitat is based on observation in the field (DJG, DSM, MW pers. obs.). See San Mauro et al. (2014), Taylor (1968), and Wilkinson, Sherratt, Starace, and Gower (2013) for more information on morphology and maternal dermatophagy.

	Rhinatrema bivittatum	*Caecilia tentaculata*	*Typhlonectes compressicauda*	*Microcaecilia unicolor*	*Microcaecilia dermatophaga*
**Habitat**	soil/litter	soil/litter	water	soil	soil
**Degree of fossoriality**	moderate	moderate	low	high	high
**Dermal fold system**	“tertiary”	primary & secondary	primary	primary & secondary	primary & secondary
**Dermal scales**	extensive	posteriorly	none	posteriorly	posteriorly
**Maternal dermatophagy**	-	likely present	-	likely present	document

Table S2. Tissue sample information and experimental design for the differential expression analysis.

Species	Number of individuals	Sex	Tissue	Sample name	SRA database number	Experimental design condition
Rhinatrema bivittatum	2	Male	Kidney	Kidney9	SRR5591443	Non-skin
			Liver	Liver9	SRR5591420	Non-skin
			Skin	Skin9	SRR5591419	Skin
			Testis	Testis9	SRR5591432	Non-skin
		Female	Foregut	Foregut79	SRR5591438	Non-skin
			Liver	Liver79	SRR5591435	Non-skin
			Lung	Lung79	SRR5591436	Non-skin
			Muscle	Muscle79	SRR5591433	Non-skin
			Skin	Skin79	SRR5591434	Skin
			Spleen	Spleen79	SRR5591442	Non-skin
Caecilia tentaculata	1	Male	Foregut	Foregut81	SRR5591452	Non-skin
			Heart	Heart81	SRR5591451	Non-skin
			Kidney	Kidney81	SRR5591454	Non-skin
			Liver	Liver81	SRR5591453	Non-skin
			Lung	Lung81	SRR5591448	Non-skin
			Muscle	Muscle81	SRR5591447	Non-skin
			Posterior skin	PosteriorSkin81	SRR5591450	Skin
			Skin	Skin81	SRR5591449	Skin
			Spleen	Spleen81	SRR5591446	Non-skin
			Testis	Testis81	SRR5591445	Non-skin
Typhlonectes compressicauda	1	Male	Foregut	Foregut83	SRR5591431	Non-skin
			Heart	Heart83	SRR5591416	Non-skin
			Kidney	Kidney83	SRR5591415	Non-skin
			Liver	Liver83	SRR5591418	Non-skin
			Lung	Lung83	SRR5591417	Non-skin
			Posterior skin	PosteriorSkin83	SRR5591444	Skin
			Skin	Skin83	SRR5591441	Skin
Microcaecilia unicolor	2	Female	Foregut	Foregut82	SRR5591426	Non-skin
			Liver	Liver82	SRR5591425	Non-skin
			Lung	Lung82	SRR5591424	Non-skin
			Muscle	Muscle82	SRR5591423	Non-skin
			Posterior skin	PosteriorSkin82	SRR5591422	Skin
			Skin	Skin82	SRR5591421	Skin
		Female	Kidney	Kidney8	SRR5591439	Non-skin
			Liver	Liver8	SRR5591440	Non-skin
			Skin	Skin8	SRR5591437	Skin
Microcaecilia dermatophaga	1	Unknown (juvenile)	Kidney	Kidney80	SRR5591430	Non-skin
			Liver	Liver80	SRR5591429	Non-skin
			Posterior skin	PosteriorSkin80	SRR5591428	Skin
			Skin	Skin80	SRR5591427	Skin

Table S3. Characterisation of significantly up-regulated sequences in the caecilian skin transcriptomes.

Uniprot ID	Gene name	Protein description	GO terms	Tissue enhanced in the Human Protein Atlas dataset	Adjusted p-value	Log_2 Fold Change
**A2VE13**	*mal2*	Multispan transmembrane protein	GO:0001766; GO:0008104; GO:0016021; GO:0016324; GO:0019911; GO:0042552; GO:0045056; GO:0045121; GO:0070062	Expressed generally	0.046814558	2.152141712
**O15231**	*znf185*	Zinc finger protein 185	GO:0005737; GO:0005856; GO:0005925; GO:0008270	Epididymis, esophagus, and seminal vesicle	0.000606255	3.325495301
**O94919**	*endod1*	Endonuclease domain-containing 1 protein	GO:0002576; GO:0003676; GO:0004519; GO:0005576; GO:0005829; GO:0016020; GO:0046872; GO:0070062	Expressed generally	0.027163336	1.882290321
**O94967**	*wdr47*	WD repeat-containing protein 47	GO:0005737; GO:0005874; GO:0007275	Expressed generally	0.004348627	1.346953179
**O95171**	*scel*	Sciellin	GO:0001533; GO:0005737; GO:0008544; GO:0009790; GO:0030216; GO:0046872; GO:0070062	Esophagus and skin	0.036957689	2.367525528
**O95377**	*gjb5*	Gap junction beta-5 protein	GO:0005922; GO:0007154; GO:0008544; GO:0016021; GO:0060707; GO:0060708; GO:0060713; GO:1905867	Esophagus and skin	0.000736441	2.818280184
**O95678**	*krt75*	Keratin, type II cytoskeletal 75	GO:0002244; GO:0005198; GO:0005829; GO:0005882; GO:0031424; GO:0045095; GO:0070062; GO:0070268	-	8.31E-05	4.739755569
**P18519**	*tnfrsf16*	Nerve growth factor receptor	GO:0005031; GO:0005516; GO:0005634; GO:0005886; GO:0005887; GO:0006919; GO:0006954; GO:0006955; GO:0007266; GO:0007411; GO:0010977; GO:0015026; GO:0031625; GO:0032496; GO:0032922; GO:0042127; GO:0042981; GO:0043005;	Expressed generally	0.001402807	1.981215918

			GO:0043121; GO:0048406; GO:0097190; GO:1900182; GO:1902895; GO:1903588			
**P31363**	*pou3f1*	POU domain, class 3, transcription factor 1-A	GO:0003700; GO:0005634; GO:0006351; GO:0006357; GO:0007420; GO:0043565	Skin	9.16E-08	4.697728521
**P34024**	*plca*	Phosphatidylinositol diacylglycerol-lyase	GO:0004436; GO:0005576; GO:0005737; GO:0008081; GO:0009405; GO:0016042	-	3.15E-07	5.946929132
**P53770**	*dlx3*	Homeobox protein DLX-3	GO:0005634; GO:0006355; GO:0007275; GO:0043565	Placenta and skin	1.48E-24	7.390548223
**P55849**	*dsc1*	Desmocollin-1	GO:0005509; GO:0005886; GO:0007156; GO:0016021; GO:0030057; GO:0070062	Skin	7.37E-05	2.948641112
**Q05004**	*nxpe1*	Brush border protein	GO:0005576	Colon, rectum	0.011153655	2.588884021
**Q09666**	*ahnak*	Desmoyokin	GO:0003723; GO:0005634; GO:0005737; GO:0005765; GO:0005829; GO:0005886; GO:0005925; GO:0015629; GO:0016020; GO:0030315; GO:0031982; GO:0042383; GO:0043034; GO:0043484; GO:0044291; GO:0044548; GO:0045296; GO:0051259; GO:0070062; GO:0097493; GO:1901385	Expressed generally	0.032811518	1.244850455
**Q10586**	*dbp*	D site-binding protein	GO:0000977; GO:0001077; GO:0001889; GO:0005634; GO:0006357; GO:0007275; GO:0007623; GO:0045944	Expressed generally	0.000636255	2.886685174
**Q1JQA4**	*tspan15*	Tetraspanin-15	GO:0005887; GO:0007166; GO:0009986; GO:0019899; GO:0031902; GO:0051604; GO:0070062; GO:0090002; GO:0097197	Expressed generally	0.002730037	1.498620555
**Q1LVW0**	*btbd11a*	Ankyrin repeat and BTB/POZ domain-containing protein BTBD11-A	GO:0000786; GO:0003677; GO:0005634; GO:0005737; GO:0016021; GO:0019005; GO:0030162; GO:0031625; GO:0042787; GO:0043161; GO:0046982; GO:0060395	Parathyroid gland	0.045919504	1.723118039
**Q2KNA0**	*speccl1*	Cytospin-A	GO:0005737; GO:0005815; GO:0005819; GO:0005921; GO:0007026; GO:0007049;	Expressed generally	0.019297852	1.798481517

			GO:0016477; GO:0030036; GO:0030835; GO:0031941; GO:0051301			
**Q2T9K2**	*tfap2e*	Transcription factor AP-2-epsilon	GO:0003677; GO:0003700; GO:0005634; GO:0006351	Skin	6.02E-06	4.440094364
**Q32MQ0**	*znf750*	Zinc finger protein 750	GO:0001046; GO:0001077; GO:0005634; GO:0005730; GO:0008544; GO:0030154; GO:0043231; GO:0045944; GO:0046872; GO:1990841	Esophagus and skin	2.44E-07	5.442262925
**Q3L254**	*wnt7b*	Protein Wnt-7b	GO:0005109; GO:0005578; GO:0005615; GO:0016055; GO:0030182; GO:0045165; GO:0070307	Skin	0.033257584	2.361343934
**Q3SWY4**	*lrrn4cl*	LRRN4 C-terminal-like protein	GO:0016021	Expressed generally	0.000121558	2.545750467
**Q3V5L5**	*mgat5b*	Alpha-1,6-mannosylglycoprotein 6-beta-N-acetylglucosaminyltransferase B	GO:0000139; GO:0005794; GO:0006487; GO:0016021; GO:0030144; GO:0046872	Cerebral cortex	0.008455543	2.979659158
**Q4VAE3**	*tmem65*	Transmembrane protein 65	GO:0003231; GO:0005739; GO:0005743; GO:0005886; GO:0014704; GO:0016021; GO:1903779	Expressed generally	0.011779563	1.036585843
**Q4VC05**	*bcl7a*	B-cell CLL/lymphoma 7 protein family member A	GO:0045892	Expressed generally	0.003519799	1.479851356
**Q4VNC1**	*atp13a4*	Probable cation-transporting ATPase 13A4	GO:0005388; GO:0005524; GO:0005886; GO:0005887; GO:0006874; GO:0019829; GO:0034220; GO:0043231; GO:0046872	Epididymis, parathyroid gland, thyroid gland	6.78E-06	4.677358853
**Q58DL7**	*arhgef9*	Collybistin	GO:0005089; GO:0005829; GO:0035023	Expressed generally	0.001172687	2.827209183
**Q5JW98**	*fam26d*	Protein FAM26D	GO:0005261; GO:0005887; GO:0034220	Placenta	0.001990421	3.279102681
**Q5M7N9**	*esyt3*	Extended synaptotagmin-3	GO:0005886; GO:0006869; GO:0008289; GO:0016021; GO:0031227; GO:0044232; GO:0046872	Lung, skin, thyroid gland	0.031547858	2.026833989
**Q5ZHQ2**	*lgalsl*	Galectin-related protein	GO:0005737; GO:0030246	Expressed generally	0.012470091	2.355960573
**Q66KX2**	*cadm4*	Cell adhesion molecule 4	GO:0007155; GO:0016021	Cerebral cortex	0.017628461	2.067869795

**Q6AZM3**	*reep4*	Receptor expression-enhancing protein 4	GO:0005783; GO:0005789; GO:0005874; GO:0006998; GO:0007084; GO:0008017; GO:0016021; GO:0051301	Expressed generally	0.02797932	1.629959292
**Q6BBL6**	*cldn4*	Claudin-4	GO:0005198; GO:0005887; GO:0005923; GO:0016327; GO:0016338; GO:0042802; GO:0061436	Expressed generally	3.08E-06	5.037737827
**Q6GR45**	*eif6*	Eukaryotic translation initiation factor 6 (eIF-6)	GO:0003743; GO:0005730; GO:0005737; GO:0042256; GO:0043022	Expressed generally	0.00028972	1.017060671
**Q6KB66**	*krt80*	Keratin, type II cytoskeletal 80	GO:0005198; GO:0005737; GO:0005829; GO:0005882; GO:0031424; GO:0045095; GO:0045111; GO:0070268	Skin	2.60E-06	4.646101698
**Q6XQH0**	*gal3st2*	Galactose-3-O-sulfotransferase 2	GO:0001733; GO:0008146; GO:0009101; GO:0009247; GO:0016020; GO:0016021; GO:0032580; GO:0050694; GO:0051923	Colon and rectum	0.046814558	2.676687744
**Q7ZYQ0**	*foxi1e*	Forkhead box protein I1-ema	GO:0003700; GO:0005634; GO:0006351; GO:0007275; GO:0042664; GO:0043565; GO:0045893; GO:0048335	-	0.015743687	2.981023402
**Q86SQ4**	*adgrg6*	Adhesion G-protein coupled receptor G6	GO:0004930; GO:0005518; GO:0005622; GO:0005886; GO:0007005; GO:0007166; GO:0007186; GO:0010579; GO:0014037; GO:0016021; GO:0019933; GO:0022011; GO:0042552; GO:0043236; GO:0050840; GO:0060347	Placenta	0.00639498	1.965019155
**Q86UK0**	*abca12*	ATP-binding cassette sub-family A member 12	GO:0005102; GO:0005319; GO:0005524; GO:0005737; GO:0005743; GO:0005829; GO:0005886; GO:0006869; GO:0010875; GO:0016021; GO:0019725; GO:0031424; GO:0032940; GO:0033700; GO:0034040; GO:0034191; GO:0035627; GO:0042626; GO:0043129; GO:0043231; GO:0045055; GO:0048286; GO:0055085; GO:0055088; GO:0061436; GO:0072659;	Breast and skin	4.05E-05	3.848213513

			GO:0097209; GO:2000010			
**Q8BW74**	*hlf*	Hepatic leukemia factor	GO:0000977; GO:0001077; GO:0001228; GO:0005634; GO:0035914; GO:0043565; GO:0045944; GO:0048511	Expressed generally	0.047379797	1.730771243
**Q8IWB1**	*itprip*	Inositol 1,4,5-trisphosphate receptor-interacting protein	GO:0005886; GO:0016020	Expressed generally	0.033439292	2.114786378
**Q8NFD2**	*ankk1*	Ankyrin repeat and protein kinase domain-containing protein 1	GO:0004674; GO:0005524	Skin	6.69E-05	3.265571376
**Q8NFQ6**	*bpifc*	BPI fold-containing family C protein	GO:0001530; GO:0005543; GO:0005615	Skin	1.62E-12	7.212949827
**Q8TCA0**	*lrrc20*	Leucine-rich repeat-containing protein 20	-	Skeletal muscle	0.013581506	2.642562647
**Q8TD16**	*bicd2*	Protein bicaudal D homolog 2	GO:0000042; GO:0005635; GO:0005642; GO:0005643; GO:0005794; GO:0005829; GO:0005856; GO:0005886; GO:0006890; GO:0017137; GO:0031410; GO:0034452; GO:0051028; GO:0051642; GO:0051959; GO:0070840; GO:0072385; GO:0072393	Expressed generally	0.00639498	1.830649916
**Q8VCA5**	*tmprss4*	Transmembrane protease serine 4	GO:0004252; GO:0005044; GO:0016021	Rectum and urinary bladder	3.42E-05	3.261649905
**Q90372**	*qnr-71*	Protein QNR-71	GO:0016021	-	0.012470091	1.830499398
**Q92754**	*tfap2c*	Transcription factor AP-2 gamma	GO:0000122; GO:0000977; GO:0001047; GO:0001077; GO:0001078; GO:0003677; GO:0003700; GO:0005634; GO:0005654; GO:0005739; GO:0005829; GO:0006357; GO:0007267; GO:0008584; GO:0040029; GO:0042127; GO:0045944; GO:0046983	Skin	7.35E-06	4.975393316
**Q96EL1**	*fam212a*	PAK4-inhibitor INKA1	GO:0005634; GO:0005737; GO:0019901; GO:0021915; GO:0030291; GO:0070062	Skin	0.036957689	2.001293676
**Q96RD7**	*panx1*	Pannexin-1	GO:0002020; GO:0002931; GO:0005102; GO:0005262;	Expressed generally	0.000615936	1.751947004

			GO:0005783; GO:0005789; GO:0005886; GO:0005921; GO:0006812; GO:0006816; GO:0007267; GO:0016020; GO:0016021; GO:0022840; GO:0032059; GO:0033198; GO:0034214; GO:0043234; GO:0044325; GO:0046982; GO:0050717; GO:0050718; GO:0051015; GO:0055077; GO:0097110			
**Q9BVG8**	*kifc3*	Kinesin-like protein KIFC3	GO:0003777; GO:0005524; GO:0005794; GO:0005813; GO:0005871; GO:0005874; GO:0005915; GO:0007018; GO:0007030; GO:0007601; GO:0008017; GO:0008569; GO:0016887; GO:0030659; GO:0045218; GO:0070062; GO:0090136	Expressed generally	0.011153655	2.86600906
**Q9BXI3**	*nt5c1a*	Cytosolic 5'-nucleotidase 1A	GO:0000166; GO:0000287; GO:0005829; GO:0006195; GO:0008253; GO:0009116; GO:0009128; GO:0046085; GO:0046135	Heart muscle and skeletal muscle	2.70E-06	1.709244848
**Q9D7I9**	*tgm5*	Transglutaminase-5	GO:0003810; GO:0005737; GO:0018149; GO:0046872	Esophagus and skin	0.005268197	3.591491589
**Q9I9M5**	*fzd1*	Frizzled-1	GO:0004930; GO:0005886; GO:0007275; GO:0016021; GO:0042813	Expressed generally	0.015121931	1.108554378
**Q9NUC0**	*sertad4*	SERTA domain-containing protein 4	GO:0005634	Expressed generally	0.048330757	2.326310075
**Q9NYQ8**	*fat2*	Protocadherin Fat 2	GO:0005509; GO:0005634; GO:0005886; GO:0005913; GO:0007156; GO:0010631; GO:0016021; GO:0070062	Skin	4.54E-10	5.281769654
**Q9UNA3**	*a4gnt*	Alpha-1,4-N-acetylglucosaminyltransferase	GO:0000139; GO:0005975; GO:0006493; GO:0008375; GO:0009101; GO:0016020; GO:0016021; GO:0016266; GO:0050680	Stomach	0.00168146	3.961880549
**Q9Y5F6**	*pcdhgc5*	Protocadherin gamma-C5	GO:0005509; GO:0005887; GO:0007155; GO:0007156; GO:0007267; GO:0007399; GO:0070062	Cerebral cortex	0.042191149	1.458789538

**Q9Y6R7**	*fcgbp*	IgGFc-binding protein	GO:0070062	Colon	0.002961722	3.951361199

Table S4. Antimicrobial peptide annotation (APD3[1], DADP[2], Uniprot terms: Andersonin[3], Cathelicidin[4], Cecropin[5] and Magainin[6]) and occurrence (indicated by an X) in skin transcriptomes of the five sampled caecilian species.

AMP ID	Gene name	Protein description	*Rhinatrema bivittatum*	*Caecilia tentaculata*	*Typhlonectes compressicauda*	*Microcaecilia unicolor*	*Microcaecilia dermatophaga*
AP00140[1]	SK84	Glycine-rich AMP		X	X	X	X
AP00208[1]	P80230	Inhibitory polypeptide	X	X	X	X	X
AP00294[1]	eNAP-1	Alpha defesin	X	X	X	X	X
AP00400[1]	P80952	Skin peptide tyrosine-tyrosine			X	X	
AP00429[1]	NK-lysin	Effector peptide of cytotoxic T/NK cells		X	X	X	
AP00481[1]	Kaliocin-1	Lactoferrin	X	X			
AP00489[1]	Hipposin	Histone-derived AMP	X	X	X	X	X
AP00536[1]	Luxuriosin	AMP with Kunitz domain				X	X
AP00612[1]	Chrombacin	Sulfated phosphorylated peptide	X	X		X	X
AP00630[1]	Amoebapore A	Saposin-like protein	X				X
AP00812[1]	Enkelytin	Proenkephalin-A neuropeptide		X		X	
AP01157[1]	Ixodidin	Cys-rich AMP	X	X	X	X	X
AP01339[1]	BHP	Hemoglobin peptide		X	X	X	
AP01340[1]	Naegleriapore A	Saposin-like protein	X	X	X		X
AP01372[1]	CXCL14	Chemokine			X		X
AP01373[1]	Thrombocidin1	Chemokine		X	X		
AP01374[1]	Thrombocidin2	Chemokine			X	X	
AP01474[1]	NPY	Neuropeptide Y	X	X			
AP01476[1]	CGRP	Calcitonin gene-related neuropeptide		X	X		
AP01477[1]	VIP	Vasoactive neuropeptide	X	X	X	X	X
AP01479[1]	Adrenomedullin	Neuropeptide	X	X	X	X	X
AP01522[1]	Ap	Antifungal peptide		X	X	X	
AP01540[1]	SP-BN	N-terminal region of surfactant protein B saposin-like	X	X	X	X	X
AP01575[1]	TCP	Thrombin-derived C-terminal peptide	X	X	X	X	X
AP01580[1]	Elafin	Elastase-specific inhibitor	X				
AP01646[1]	gcLEAP-2	Grass carp liver-expressed antimicrobial peptide-2		X			
AP01676[1]	Abeta42	Beta-amyloid	X	X	X	X	X

		peptide					
AP02012[1]	YFGAP	Yellowfin tuna GAPDH-related AMP	X	X	X	X	X
AP02017[1]	hGAPDH(2-32)	Fragment of glyceraldehyde-3-phosphate dehydrogenase		X			
AP02030[1]	cgUbiquitin	Hemolytic peptide	X	X	X	X	X
AP02068[1]	Ang1	Murine angiogenin 1			X		
AP02069[1]	Ang4	Murine angiogenin 4	X				
AP02070[1]	RegIIIgamma	Secreted C-type lectin AMP	X	X	X	X	X
AP02071[1]	RegIIIalpha	Secreted C-type lectin AMP	X	X	X	X	X
AP02075[1]	CCL20	Macrophage inflammatory protein-3alpha	X				
AP02076[1]	CXCL1	Chemokine		X	X	X	
AP02078[1]	CXCL3	Chemokine	X	X	X	X	X
AP02080[1]	CXCL10	Chemokine		X	X	X	X
AP02081[1]	CXCL11	Chemokine		X			
AP02082[1]	CXCL12	Chemokine			X		
AP02083[1]	CXCL13	Chemokine		X	X	X	
AP02084[1]	XCL1	Lymphotactin chemokine	X	X	X		
AP02087[1]	CCL11	Eotaxin	X				
AP02088[1]	CCL13	Chemokine	X				
AP02090[1]	CCL18	Chemokine				X	X
AP02091[1]	CCL19	Chemokine	X		X		X
AP02092[1]	CCL25	Chemokine	X	X			
AP02095[1]	SLPI	Secretory leukocyte protease inhibitor	X	X	X	X	X
AP02096[1]	UBI	Murine peptide	X	X	X	X	X
AP02122[1]	pCM1	Fragment of hglyrichin	X				
AP02185[1]	CXCL6	Chemokine	X	X			X
AP02186[1]	CCL28	Chemokine	X	X	X	X	
AP02188[1]	mCCL28	Chemokine	X	X			X
AP02195[1]	Chemerin	Retinoic acid receptor responder protein 2	X	X	X	X	X
AP02230[1]	HMGN2	High mobility group nucleosomal binding domain 2	X	X	X		X
AP02257[1]	Lysozyme	Lectin-binding enzyme	X	X	X	X	X
2965[2]	Buforin 2	Histone H2A derived AMP	X	X	X	X	X
P80952[2]	-	Skin peptide tyrosine-tyrosine	X	X	X	X	
P83578[2]	-	Proteinase inhibitor PSKP-1		X			
P86282[2]	Phylloseptin Bu-1	-	X		X	X	X
D2K8I9[3]	-	Andersonin-9 AMP	X	X	X	X	X
E3SZM1[3]	-	Andersonin-8a		X			

		peptide					
E3SZM2[3]	-	Andersonin-8b peptide	X		X	X	X
E3SZM5[3]	-	Andersonin-11 peptide	X	X	X	X	X
E3SZM6[3]	-	Andersonin-7 peptide			X	X	
K7GIB1[4]	-	Uncharacterized protein cathelicidin-like			X		
A0A067QGP8[4]	L798_02828	Cathelicidin-B1	X				X
A0A096N3S1[4]	-	Uncharacterized protein cathelicidin-like	X	X	X	X	X
D3ZMP7[4]	LOC689081	Cystatin	X				
F7C0D9[4]	KNG1	Kininogen 1	X		X		X
F7C0Z0[4]	KNG1	Kininogen 1				X	
G1PML8[4]	KNG1	Kininogen 1				X	
G3VCA4[4]	CST7	Cystatin					X
G3VCA5[4]	CST7	Cystatin			X	X	
H3A036[4]	CST7	Cystatin	X	X	X	X	X
I3IVB6[4]	-	Uncharacterized protein cathelicidin-like	X				
I3JY77[4]	LOC100708655	Fetuin B	X	X		X	X
K7FKZ8[4]	-	Uncharacterized protein cathelicidin-like					X
K7FPA2[4]	-	Uncharacterized protein cathelicidin-like				X	
K7GI21[4]	-	Uncharacterized protein cathelicidin-like	X		X	X	X
K7GID5[4]	-	Uncharacterized protein cathelicidin-like	X		X	X	X
M7BBJ0[4]	UY3_13360	Uncharacterized protein cathelicidin-like	X			X	X
Q5M8F3[4]	cst7	Cystatin		X		X	X
R0LBE5[4]	Anapl_03194	Kininogen 1				X	
P82115[5]	prtA	Serralysin	X	X	X		X
Q94527[5]	Rel CG11992	Nuclear factor NF-kappa-B p110 subunit	X	X	X	X	X
B4R1J7[5]	Anp	Andropin	X	X	X	X	X
Q589Y5[5]	ohsp1	Serine protease	X	X	X	X	X
Q7PT80[5]	REL2 1270310	AGAP006747-PA	X	X	X	X	X
P05223[6]	-	Preprocaerulein type I	X				
Q45TR8[6]	-	Ubiquitin	X	X	X	X	X

Table S5. Peptide pheromones annotation and occurrence (indicated by an X) in skin transcriptomes of the five sampled caecilian species

Pheromone ID	Gene name	Protein description	*Rhinatrema bivittatum*	*Caecilia tentaculata*	*Typhlonectes compressicauda*	*Microcaecilia unicolor*	*Microcaecilia dermatophaga*
A0A0A0QT03	Cloa_05	Sodefrin-like factor beta isoform 05	X				
A0A0A0QTC2	Cloa_19	Sodefrin-like factor alpha isoform 19				X	
A0A0A0QTD8	Cloa_02	Sodefrin-like factor beta isoform 02		X			
A0A0A0QU60	Cloa_08	Sodefrin-like factor beta isoform 07	X				
A0A0A0QU84	Cloa_26	Sodefrin-like factor beta isoform 24				X	
A0A0A0QUZ5	Cloa_10	Sodefrin-like factor beta isoform 10		X			
A0A0A0QVR4	Cloa_09	Sodefrin-like factor alpha isoform 09	X	X		X	
A0A0A0QVU0	Cloa_05	Sodefrin-like factor beta isoform 04		X			
A0A0A0QVV1	Cloa_30	Sodefrin-like factor alpha isoform 28					X
A0A0A0QVV6	Cloa_01	Sodefrin-like factor beta isoform 01	X				X
A0A0B5GR37	-	Sodefrin-like factor 9	X				
A0A0B5H1E4	-	Sodefrin-like factor 1	X				
A0A0B5H3N9	-	Sodefrin-like factor 20		X			
A0A0B5H6P8	-	Sodefrin-like factor 15				X	X
A0A0E3KK02	SPF	Sodefrin-like factor	X		X	X	X
A0A0E3KK06	SPF	Sodefrin-like factor					X
A0A0E3N0I6	SPF	Sodefrin-like factor			X		
A0A0E3N2L5	SPF	Sodefrin-like factor	X	X		X	
A0A0E3N2L9	SPF	Sodefrin-like factor		X			
A0A0E3N3L3	SPF	Sodefrin-like factor		X			
A0A0E3N4Q2	SPF	Sodefrin-like factor		X			
A0A0F7JG78	-	Sodefrin-like factor			X		
A0A0F7JHQ7	-	Sodefrin-like factor			X		
A0A0F7JHR5	-	Sodefrin-like factor					X

A0A0F7JJU1	-	Sodefrin-like factor				X	
A0A125S9K3	SPF1	Sodefrin-like factor	X				
A0A125S9K5	SPF8	Sodefrin-like factor			X	X	X
A0A125S9K7	SPF2	Sodefrin-like factor				X	X
A0A125S9K8	SPF7	Sodefrin-like factor				X	
A0A125S9L0	SPF4	Sodefrin-like factor	X				
A0A125S9L4	SPF10	Sodefrin-like factor			X		
A0A125S9L5	SPF11	Sodefrin-like factor					X
A0A140IHG1	-	Sodefrin-like factor					X
A0A140IHG6	-	Sodefrin-like factor			X		X
A0A140IHH1	-	Sodefrin-like factor			X		
A0A140IHH2	-	Sodefrin-like factor		X			
A0A172AZC5	-	Sodefrin-like factor			X		
B2CM93	-	Sodefrin-like factor			X		
G3IMK8	I79_025148	Aphrodisin		X			
Q2EFD2	-	Sodefrin-like factor	X				
Q4FAD1	SPF1	Sodefrin-like factor				X	
Q4FAE1	SPF1	Sodefrin-like factor				X	
Q4FAG1	SPF1	Sodefrin-like factor					X

Table S6. Annotated AMPs and p-values for Wilcoxon signed-rank test of differences between AMP expression levels in midbody and posterior skin samples. * indicates self-built databases for subset of entries for these UniProt terms (see Materials and Methods). For *R. bivittatum,* data were available only for midbody skin.

**Database**	***Rhinatrema bivittatum***	***Caecilia tentaculata***	***Typhlonectes compressicauda***	***Microcaecilia unicolor***	***Microcaecilia dermatophaga***
**APD3**	36	41	37	32	30
**DADP**	3	3	3	3	2
**Andersonin***	3	3	4	4	3
**Cathelicidin***	10	4	7	12	11
**Cecropin***	5	5	5	4	5
**Magainin***	2	1	1	1	1
**p-value**	-	1.31e-14	1.551e-12	< 2.2e-16	< 2.2e-16

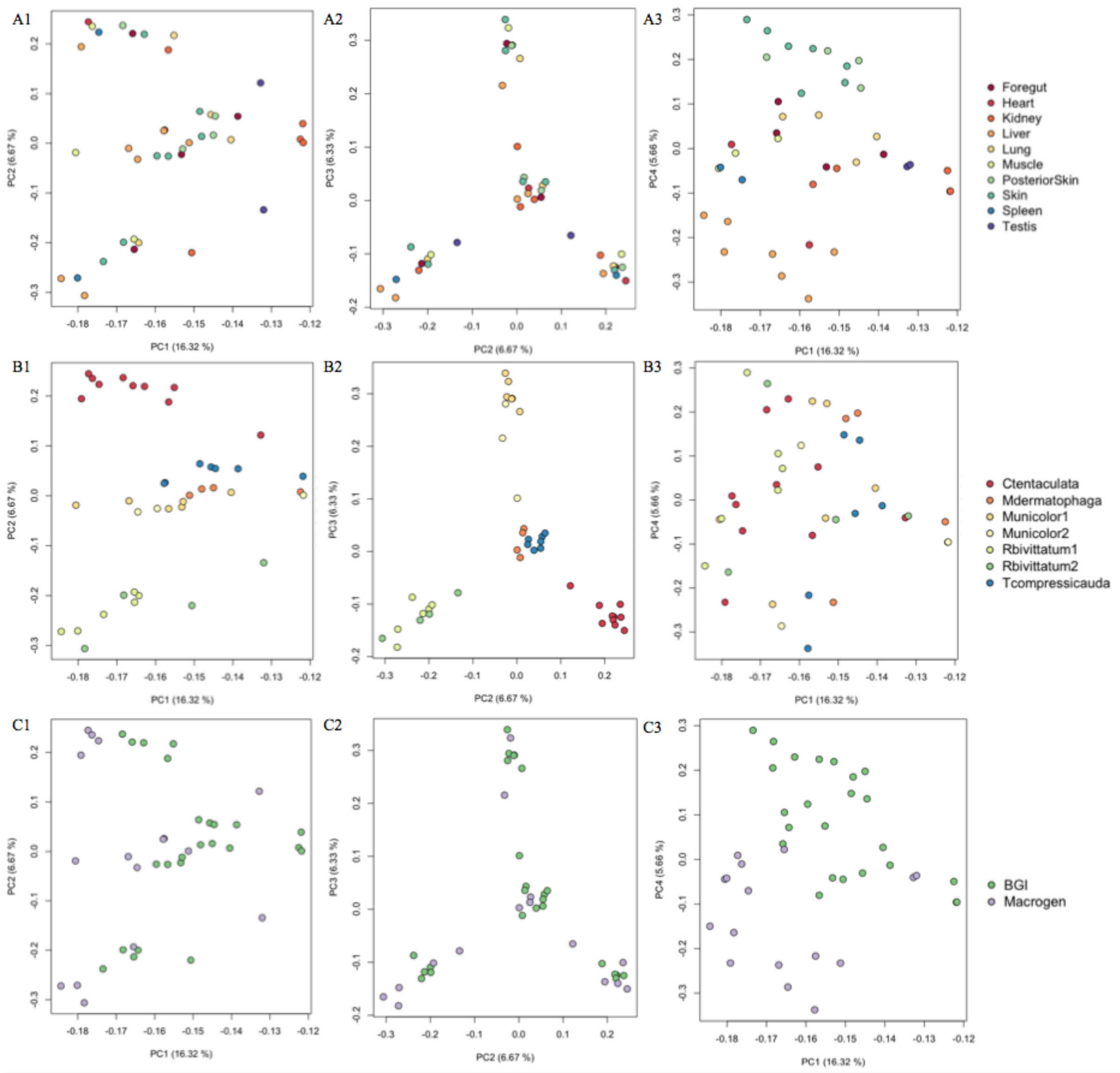

Figure S1. PCA plots of PC1 versus PC2 (first column), PC2 versus PC3 (second column), and PC1 versus PC4 (third column) showing variance among gene expression levels in various tissue types across the five sampled caecilian species. Samples were color coded by tissue type (first row), species (second row), and sequencing company (third row).

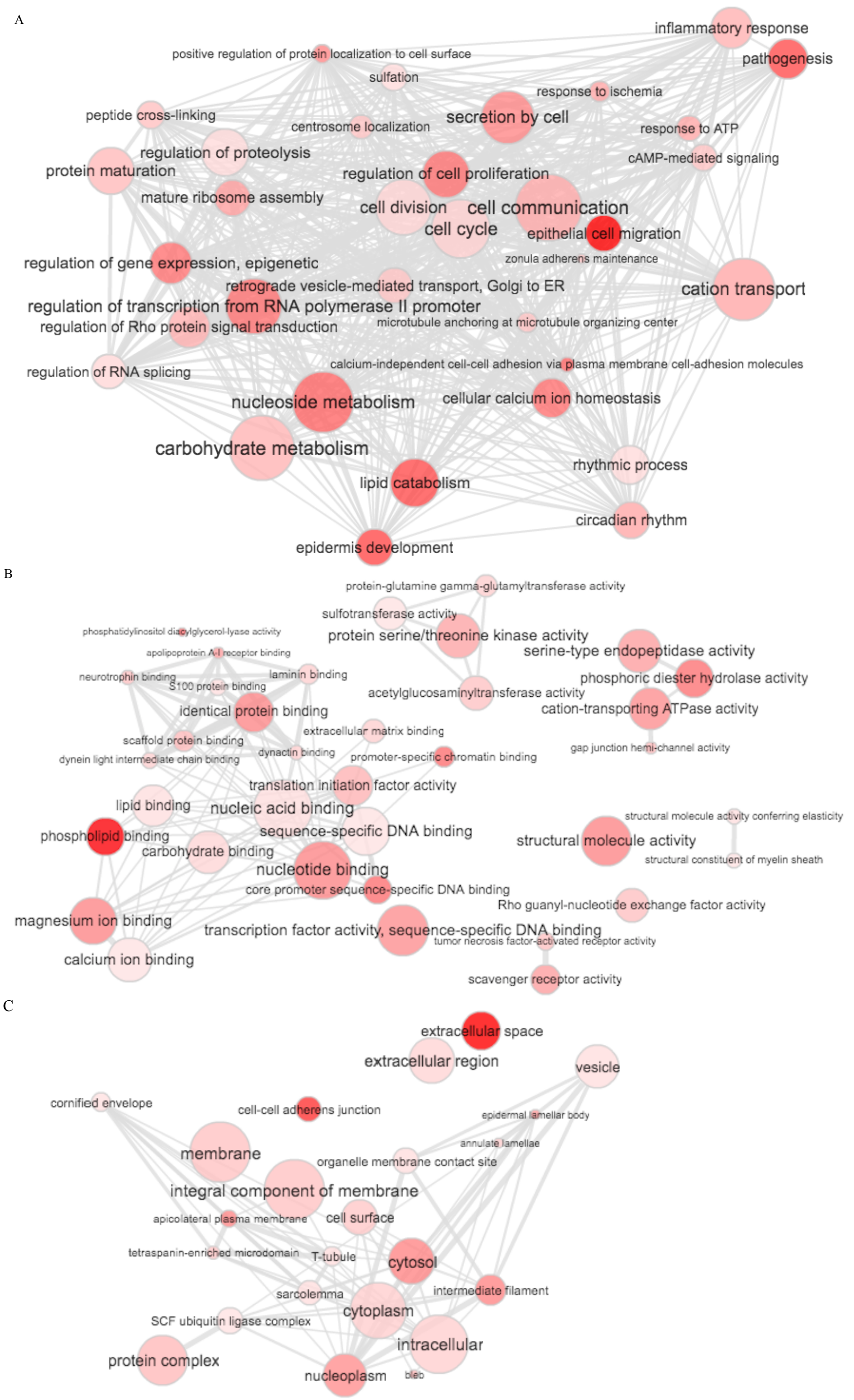

Figure S2. Network graphs for GO domains (A: biological process, B: molecular function and C: cellular component) of skin up-regulated genes. Greater colour intensity indicates higher fold change in expression in caecilian skin, and circle size is positively correlated with number of genes with the same GO in the UniProt database.

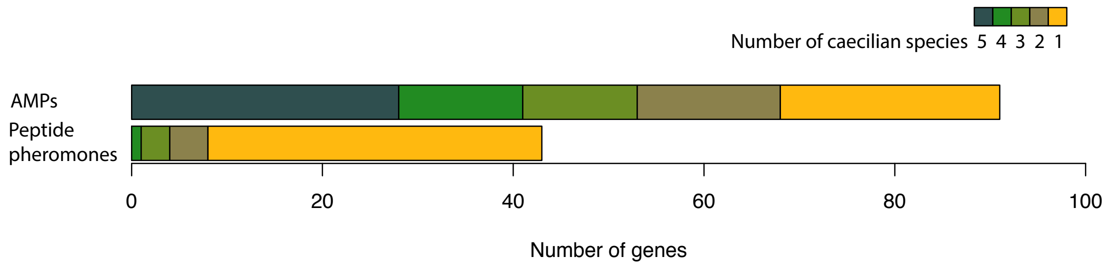

Figure S3. Expressed sequences annotated as encoding peptides and their presence in the skin of the five sampled caecilian species.
